# The correlation between high-sensitivity cardiac troponin levels in diabetic patients’ serum and lower limb lesions: based on NHANES data

**DOI:** 10.3389/fendo.2025.1515212

**Published:** 2025-01-27

**Authors:** Lin Li, Linxi Jiao, Danyang Yang, Jingxia Zhao, Ping Li

**Affiliations:** Beijing Hospital of Traditional Chinese Medicine, Capital Medical University, Beijing Institute of Traditional Chinese Medicine, Beijing, China

**Keywords:** hypersensitive cardiac troponin, diabetes mellitus, lower extremity disease, peripheral neuropathy, peripheral vascular disease

## Abstract

**Objective:**

To evaluate the association of hypersensitive cardiac troponin (hs-cTnT), a biomarker of myocardial injury, with diabetic lower extremity disease in American adults.

**Methods:**

We conducted a cross-sectional study (unweighted N=1,196) of diabetic patients aged 40 years or older who participated in the National Health and Nutrition Examination Survey (NHANES) from 1999 to 2004. Logistic regression was used to assess the association of hs-cTnT with lower extremity disease, including peripheral neuropathy (as assessed by monofilament test), peripheral artery disease (as assessed by ankle-brachial index), history of foot ulcers, or amputation. All analyses are weighted.

**Results:**

The prevalence rate of diabetic lower extremity disease was 41.6%. Adjusted hs-cTnT was significantly associated with lower limb disease in adults with diabetes. There is interaction between chronic kidney disease and hs-cTnT, which strongly interferes with the correlation between hs-cTnT and lower extremity lesions in diabetic patients.

**Conclusions:**

Our study suggests the usefulness of hs-cTnT as a biomarker for lower extremity lesions in adults with diabetes and highlights the potential direct interaction of hs-cTnT with chronic kidney disease.

## 1 Introduction

Lower limb diseases include peripheral neuropathy (PN) or peripheral vascular disease (PAD), which can lead to foot ulcers, amputations, and death (1). The prevalence of lower limb diseases is closely related to the status of diabetes, and studies have shown that the prevalence of PAD and PN in people with diabetes is about twice that of the general population (2). Approximately 50% of diabetic patients will develop foot ulcers, and diabetes is the main cause of lower limb amputations (3).

High-sensitivity cardiac troponin (hs-cTnT) is an important marker for assessing myocardial injury (4). It provides information about myocardial injury by measuring the levels of cardiac troponin in the blood. This new high-sensitivity measurement technology allows for the detection of smaller myocardial injuries, thereby increasing the sensitivity and diagnostic accuracy of myocardial injury (5). Studies have shown that diabetic patients often have an increased risk of heart disease and myocardial injury (6). The levels of hs-cTnT in diabetic patients are closely related to the risk of cardiovascular diseases (7). Therefore, the measurement of hs-cTnT can help doctors assess the cardiovascular risk of diabetic patients and take appropriate preventive and treatment measures (8). However, few studies have investigated the link between hs-cTnT and diabetic lower limb diseases. This study aims to investigate the correlation between hs-cTnT and lower limb diseases in diabetic patients in the United States.

## 2 Subjects and methods

### 2.1 Study population and design

The National Health and Nutrition Examination Survey (NHANES) is a crucial research program that aims to evaluate the health and nutritional status of both adults and children residing in the United States. The Centers for Disease Control and Prevention (CDC) is responsible for furnishing health statistics for the nation, and the protocols of NHANES have been duly approved by the Research Ethics Review Board of NCHS. To ensure the protection of the participants’ rights, NHANES has obtained informed written consent from all the individuals involved in the study. Moreover, the datasets generated and analyzed in the current study are readily available on the official NHANES website (https://www.cdc.gov/nchs/nhanes/index.html). We downloaded data of NHANES from 1999 to 2004.Individuals were considered to have diabetes if they met one of the following criteria: (1) previously diagnosed with diabetes by a physician; (2) glycated hemoglobin (HbA1c) level ≥6.5% (48 mmol/mol); (3) fasting blood glucose level ≥126 mg/dL; (4) random blood glucose ≥200 mg/dL; (5) use of insulin or oral hypoglycemic agents. The following populations were excluded: (1) individuals under 40 years of age; (2) pregnant women; (3) those without information on blood high-sensitivity cardiac troponin (hs-cTnT) levels or lower limb disease. Ultimately, 1196 diabetic patients were included in the study.

### 2.2 Serum hs-cTnT

The hs-cTnT content in serum samples from NHANES 1999-2004 was measured using the Roche Cobas e601 analyzer. The lower limit of detection (LOD) is 3 ng/L and the 99th URLs is 22 ng/L for males and 14 ng/L for females.

### 2.3 Lower limb disease

The primary outcome was the presence of any lower limb disease, including peripheral neuropathy (PN), peripheral arterial disease (PAD), or a self-reported history of non-healing foot ulcers, observed foot ulcers, or minor or major amputations. PN and PAD were assessed by trained researchers at mobile examination centers using standardized protocols (9). PN was evaluated using the standard 10-g monofilament test, with a 5.07 Semmes-Weinstein nylon monofilament attached to a plastic handle. All participants underwent monofilament testing at three sites on each foot, and PN was defined as at least one insensate site on each foot. Ankle-brachial pressure index (ABI) was calculated by measuring systolic blood pressure in the right brachial artery and both posterior tibial arteries using Doppler ultrasound. If participants were unable to have their blood pressure measured in the right arm, the systolic blood pressure of the left brachial artery was used instead. PAD was defined as an ABPI of less than 0.9 on the right or left side. PN and PAD were analyzed as separate outcomes.

### 2.4 Covariates

All covariate information was obtained by trained researchers following standardized procedures according to NHANES protocols (10). Age, gender, race/ethnicity, education level, smoking status, and medication use in the last month were self-reported by participants. Body mass index (BMI) was calculated as measured weight (kilograms) divided by the square of measured height (meters). Hypertension was defined as self-reported physician-diagnosed hypertension. Hypercholesterolemia was defined as self-reported physician-diagnosed hypercholesterolemia. Chronic kidney disease was defined as eGFR (estimated glomerular filtration rate) <60 mL/min per 1.73 m^2^ or a urinary albumin-to-creatinine ratio ≥ 30 mg/g.

### 2.5 Statistical analysis

Participants were categorized into quartiles (Q1-Q4) based on serum hs-cTnT levels and underwent both overall and stratified analyses. Characteristics of the overall study population were examined according to the categorization of hs-cTnT protein. Chi-square tests and analysis of variance were used to categorize variables and continuous variables for lower limb disease status, respectively. A multivariate logistic regression model was used to assess the relationship between hs-cTnT and lower limb disease outcomes. Model 1 included age, gender, and race/ethnicity. Model 2 adjusted for all variables in Model 1 as well as education level, BMI, smoking status, hypertension, and hypercholesterolemia. Model 3 further adjusted for chronic kidney disease on the basis of Model 2. The relationship between serum hs-cTnT levels and lower limb disease status was observed after stratification by chronic kidney disease.

All analyses incorporated sampling weights to obtain unbiased estimates from the complex NHANES design. The results of this study are representative of diabetic patients aged 40 or older in the United States. Analysis was conducted using SPSS, version 26 (IBM Corp), and a P-value less than 0.05 was considered statistically significant.

## 3 Results

### 3.1 Characteristics of hs-cTnT categorization in diabetic patients aged 40 and above in the United States

As shown in **Table 1**, this study included 1196 diabetic patients from NHANES (weighted to represent 34,651,362 individuals, with an average age of 60.7 years and 53% male). The prevalence of diabetic lower limb disease was 41.6% (weighted to represent 11,908,367 individuals), with a prevalence of peripheral arterial disease (PAD) at 10.7% (weighted to represent 2,650,574 individuals) and peripheral neuropathy (PN) at 26.5% (weighted to represent 8,399,960 individuals).

**Table 1:** Characteristics of hs-cTnT Categorization in Diabetic Patients Aged 40 and Above in the United States, NHANES 1999-2004.

Indicator	Overall (n=1196, N=34651362)	Human High-Sensitivity Cardiac Troponin Grouping				*P*
		Q1: hs-cTnT ≤ 5.85 ng/L (n=243,N=8634323)	Q2: hs-cTnT 5.86-9.29 ng/L (n=273,N=8592497)	Q3: hs-cTnT 9.30-15.07 ng/L (n=311,N=8783990)	Q4: hs-cTnT>15.08ng/L (n=369,N=8640553)	
Age, years	60.7 ± 11.3	52.5 ± 8.2	59.0 ± 10.8	64.1 ± 10.5	66.9 ± 9.9	<0.0001
Male, %	635 (18210896, 52.6%)	72 (3047789,35.3%)	135 (4609331,53.6%)	174 (4801432,54.7%)	254 (5752344,66.6%)	<0.0001
Ethnicity, %						
Mexican American	369 (2444100, 7.1%)	93 (789683, 9.1%)	94 (632440, 7.4%)	85 (530914, 6.0%)	97 (491063, 5.7%)	<0.0001
Other Hispanic	51 (2185575, 6.3%)	18 (858860, 9.9%)	16 (624509, 7.3%)	11 (406813, 4.6%)	6 (295393, 3.4%)	
Non-Hispanic White	469 (23243172, 67.1%)	78 (5350789, 62.0%)	97 (5664312, 65.9%)	145 (6437131, 73.3%)	149 (5790940, 67.0%)	
Non-Hispanic Black	262 (4474383, 12.9%)	42 (887664, 10.3%)	53 (1001240, 11.7%)	62 (1114615, 12.7%)	105 (1470864, 17.0%)	
Other Races – Including Multiracial	45 (2304132, 6.6%)	12 (747328, 8.7%)	13 (669996, 7.8%)	8 (294516, 3.4%)	12 (592292, 6.9%)	
Education Level, %						
Below High School	580 (11117143, 32.1%)	114 (2363381,27.4%)	132 (2456481, 28.6%)	152 (3168800, 36.1%)	182 (3128481, 36.2%)	<0.0001
High School	250 (8896443, 25.7%)	49 (2152482, 24.9%)	59 (2376635, 27.7%)	70 (2406498, 27.4%)	72 (1960828, 22.7%)	
University or Above	365 (14624519, 42.2%)	80 (4118459, 47.7%)	81 (3746125, 43.6%)	89 (3208692, 36.5%)	115 (3551243, 41.1%)	
Missing	1 (13256,0.0%)	0 (0,0.0%)	1 (13256, 0.2%)	0 (0,0.0%)	0 (0,0.0%)	
Smoking Status, %						
Non-smoker	437 (12229079, 35.3%)	61 (2225368, 57.9%)	95 (2861897, 59.8%)	133 (3703012, 72.1%)	148 (3438802, 68.4%)	<0.0001
Smoker	207 (6558421, 34.9%)	45 (1617591,42.1%)	54 (1922719,40.2%)	52 (1431404,27.8%)	56 (1586707,31.5%)	
BMI, %						
≤25kg/m^2^	181 (5075795, 14.7%)	29 (1004364, 11.9)	50 (1554717, 18.3%)	39 (1089738, 12.5%)	63 (1426974, 17.1%)	<0.0001
25-30kg/m^2^	406 (10294237, 29.7%)	86 (2608127, 35.4%)	98 (2781000, 35.9%)	114 (2531772, 36.7%)	108 (2373341, 29.3%)	
>30 kg/m^2^	557 (18013077, 52.0%)	124 (4757572, 51.0%)	118 (3999807, 43.2%)	151 (4994040, 48.6%)	164 (4261666, 44.4%)	
Hypertension, %	774 (21904624, 63.2%)	118 (4213058,48.8%)	167 (5017274,58.4%)	220 (6176624,70.3%)	269 (6497668,75.2%)	<0.0001
Hypercholesterolemia, %	600 (19008138, 59.3%)	111 (4336344,54.1%)	151 (4773099,59.3%)	165 (5151810, 63.4%)	173 (4746885,60.3%)	<0.0001
Antihyperlipidemic agents	122 (4449304,12.9%)	23 (1046633, 12.1%)	28 (1300189, 15.2%)	44 (1307508, 14.9%)	27 (794974, 9.2%)	<0.0001
Antihypertensive agents	40 (1234844, 3.6%)	11 (473019, 5.5%)	7 (199562, 2.3%)	10 (290301, 3.3%)	12 (271962, 3.2%)	<0.0001
coagulationmodifiers	33 (718656, 2.1%)	2 (60904, 0.7%)	6 (101402, 1.2%)	9 (204306, 2.3%)	16 (352044, 4.1%)	<0.0001
Chronic Kidney Disease, %	184 (3886224, 33.7%)	31 (973690, 25.1%)	28 (446249, 16.4%)	45 (1149874,41.2%)	80 (1316411, 61.8%)	<0.0001
Creatinine, Urine (mg/dL)	119.3 ± 76.1	114.0 ± 86.2	117.6 ± 58.6	115.2 ± 74.8	126.2 ± 81.8	<0.0001
Creatinine (mg/dL)	0.87 ± 0.80	1.54 ± 1.65	0.80 ± 0.25	0.72 ± 0.19	0.66 ± 0.15	<0.0001
Lower Limb Disease, %	447 (11908367, 41.6%)	56 (1726395, 22.8%)	81 (2365800, 33.4%)	119 (2972910,42.0%)	191 (4843262,70.4%)	<0.0001
Peripheral Neuropathy, %	307 (8399960, 26.5%)	39 (1113801, 13.9%)	59 (1849762, 23.6%)	74 (1969987, 23.9%)	135 (3466410,46.1%)	<0.0001
Peripheral Arterial Disease, %	118 (2650574, 10.7%)	7 (106058, 1.5%)	20 (463930, 7.1%)	38 (834775, 13.0%)	53 (1245811,26.2%)	<0.0001

**Figure 1:**
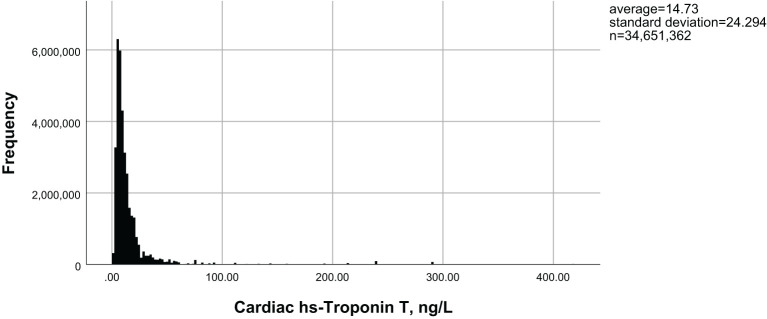
Distribution of hs-cTnT in diabetic patients aged 40 years and above in the United States.

**Figure 2:**
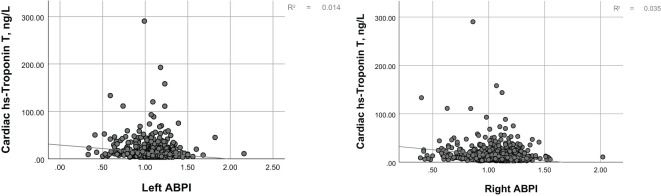
The correlation between hs-troponin T values and the ankle-brachial index.

**Figure 1** illustrates the distribution of hs-cTnT in the diabetic population over 40 years of age. Among adults with diabetes, as the levels of hs-cTnT (0.84 ng/L to 417.1 ng/L) increased, the overall prevalence of lower limb disease, lower limb PN, and lower limb PAD also increased (22.8% to 70.4%, 13.9% to 46.1%, 1.5% to 26.2%, respectively, P<0.0001). The criteria for PAD diagnosis is an ABI of less than 0.9 on either the left or right side. To further demonstrate the correlation between hs-cTnT and PAD, we performed a Pearson correlation analysis between hs-cTnT and ABI, which showed a significant correlation between the two, and we subsequently fitted a linear plot as shown in **Figure 2**. Diabetic patients with higher hs-cTnT levels were older (52.5 to 66.9, P<0.0001), more likely to be male (35.3% to 66.6%, P<0.0001), had a higher proportion of non-Hispanic blacks (10.3% to 17.0%, P<0.0001), had a higher proportion with education levels below high school (27.4% to 36.2%, P<0.0001), and had a higher risk of accompanying hypertension and hypercholesterolemia (48.8% to 75.2%, 54.1% to 60.3%, respectively, P<0.0001).

### 3.2 Association between high-sensitivity cardiac troponin (hs-cTnT) and Lower limb disease in diabetic patients aged 40 and above in the United States

In the demographically adjusted model, the percentile equivalent categories of hs-cTnT in diabetic patients were correlated with lower limb disease, peripheral neuropathy (PN), and peripheral arterial disease (PAD) (**Table 2**, Model 1). After adjustment for education level, BMI, smoking status, hypertension, and hypercholesterolemia, the correlations remained, with a trend towards a stronger association with lower limb disease and PN, and a weaker association with PAD (**Table 2**, Model 2). Further adjustment for chronic kidney disease led to a significant change in the association between hs-cTnT and lower limb disease in diabetic patients, with a downward trend in lower limb disease associated with hs-cTnT concentrations above 9.3 ng/L. Notably, the incidence of PN was lower than that corresponding to hs-cTnT concentrations below 5.85 ng/L, indicating that the trend of increasing PN incidence with increasing hs-cTnT concentrations is disrupted. Conversely, the association between hs-cTnT and PAD in diabetic patients became more pronounced (**Table 2**, Model 3). To further demonstrate the correlation between hs-cTnT and PAD, we performed a Pearson correlation analysis between hs-cTnT and ABI, which showed a significant correlation between the two, and we subsequently fitted a linear plot as shown in **Figure 2**.

**Table 2:** Association between hs-cTnT and lower limb disease in diabetic patients aged 40 and above in the United States (OR, 95% CI), NHANES 1999-2004.

	Lower Limb Disease			PN			PAD		
	Model 1	Model 2	Model 3	Model 1	Model 2	Model 3	Model 1	Model 2	Model 3
Hs-cTnT									
≤5.85 ng/L	1 (Ref)	1 (Ref)	1 (Ref)	1 (Ref)	1 (Ref)	1 (Ref)	1 (Ref)	1 (Ref)	1 (Ref)
5.86 - 9.29 ng/L	1.385 (1.382, 1.389)	1.558 (1.551, 1.564)	3.026 (2.996, 3.056)	1.586 (1.582, 1.591)	2.104 (2.094, 2.114)	1.190 (1.178, 1.203)	3.580 (3.555, 3.605)	2.505 (2.483,2.527)	17.593 (16.948, 18.261)
9.30 - 15.07ng/L	1.788 (1.783, 1.792)	2.359 (2.350, 2.369)	1.608 (1.592, 1.624)	1.478 (1.474, 1.482)	2.205 (2.195, 2.216)	0.302 (0.299, 0.306)	5.796 (5.757, 5.835)	5.607 (5.559,5.655)	22.385 (21.570, 23.231)
>15.08 ng/L	5.505 (5.491, 5.520)	7.607 (7.574, 7.640)	2.826 (2.795, 2.858)	3.654 (3.643, 3.664)	7.974 (7.935,8.013)	0.859 (0.849, 0.870)	13.490 (13.397, 13.582)	9.700 (9.613,9.788)	56.605 (54.547, 58.741)

Model 1: Age, sex, and race/ethnicity.

Model 2: All variables in Model 1 plus education level, BMI, smoking status, hypertension, hypercholesterolemia, antihyperlipidemic agents, antihypertensive agents, coagulationmodifiers.

Model 3: All variables in Model 2 plus chronic kidney disease.

### 3.3 Association between hs-cTnT and lower limb disease in diabetic patients aged 40 and above in the united states stratified by chronic kidney disease

To further verify the confounding effect of chronic kidney disease on the association between hs-cTnT and lower limb disease in diabetic patients, stratified chi-square tests were conducted in this section. The results, as shown in **Table 3**, indicate that regardless of the presence of chronic kidney disease, there is a significant positive correlation between serum hs-cTnT and lower limb disease, PN, or PAD in diabetic patients (P<0.0001). Moreover, the heterogeneity of OR before and after stratification by chronic kidney disease suggests an interaction between chronic kidney disease and hs-cTnT, the specific effects of which require further investigation. Notably, the OR value for the association between hs-cTnT and PAD in diabetic patients with chronic kidney disease is over twice that of diabetic patients without chronic kidney disease, consistent with the changes in OR values observed in the multivariate regression analysis in 2.2, confirming that chronic kidney disease strongly interferes with the association between hs-cTnT and lower limb disease in diabetic patients aged 40 and above in the United States.

**Table 3:** The impact of chronic kidney disease on the association between hs-cTnT and lower limb disease (OR, 95% CI), NHANES 1999-2004.

Hs-cTnT		Lower Limb Disease		*P*	OR Heterogeneity Test	PN		*P*	OR Heterogeneity Test	PAD		*P*	OR Heterogeneity Test
		not	yes			not	yes			not	yes		
Chronic Kidney Disease	Not	1 (Ref)	1.784 (1.780, 1.788)	<0.0001	OR Heterogeneity	1 (Ref)	1.360 (1.356, 1.364)	<0.0001	OR Heterogeneity	1 (Ref)	2.920 (2.901,2.939)	<0.0001	OR Heterogeneity
	Yes	1 (Ref)	1.640 (1.636, 1.645)	<0.0001		1 (Ref)	1.352 (1.347, 1.356)	<0.0001		1 (Ref)	6.333 (6.273,6.392)	<0.0001	

## 4 Discussion

Lower limb diseases, such as peripheral neuropathy (PN) and peripheral arterial disease (PAD), are significant complications of diabetes mellitus (11). In this study, we evaluated the relationship between hs-cTnT and lower limb disease in a nationally representative sample of diabetic patients in the United States. We found a significant association between this protein and lower limb disease in adult diabetic patients. Therefore, serum hs-cTnT may serve as a biomarker for the comorbid lower limb disease in diabetic patients. However, the association of this protein with lower limb disease in adults without diabetes warrants further investigation.

Our findings highlight a significant association between hs-cTnT levels and diabetic lower limb disease, which may reflect poor overall cardiovascular health, including manifestations of chronic lower limb ischemia. Although hs-cTnT is a sensitive indicator of myocardial injury, its specific role in diabetic lower limb disease requires further research to clarify. Few studies have examined the link between hs-cTnT and lower limb disease. Our study is pioneering in directly comparing the relationship between hs-cTnT and lower limb disease in adult diabetic patients. Previous studies have shown that hs-cTnT is independently associated with PAD or PN, regardless of the presence of diabetes, and cardiac biomarkers provide prognostic information for mortality in PAD and PN status (12–15). In the current study, we confirmed a strong association between hs-cTnT and lower limb disease in adult diabetic patients, particularly PN and PAD. hs-cTnT, a marker of myocardial injury, is primarily used for the early diagnosis and assessment of cardiac diseases such as acute coronary syndrome and myocardial infarction (16). However, even after removing cardiovascular risk factors such as hypertension, the positive correlation between hs-cTnT levels and the incidence of diabetic lower limb disease remains, and even shows an increasing trend compared to before removal. This suggests that hs-cTnT is an independent risk factor for diabetic lower limb disease with potential predictive value for lower limb lesions. Long-term accumulation of hyperglycemia can cause various injuries, and randomized controlled trials have shown that intensive glycemic control is important for the long-term prevention of macrovascular diseases (17). Therefore, the similar role of hs-cTnT in lower limb disease without diabetes warrants further investigation.

The study also found that chronic kidney disease strongly interferes with the association between hs-cTnT and lower limb disease in diabetic patients, indicating an interaction between hs-cTnT and chronic kidney disease in diabetic patients, suggesting a potential role of hs-cTnT in diabetic chronic kidney disease. Studies have found that patients with chronic kidney disease often have concurrent acute myocardial infarction, and the incidence of type 1 myocardial infarction in patients with kidney damage is increased by 1 time, while the incidence of type 2 myocardial infarction and non-ischemic myocardial injury is increased by 3 to 4 times, some studies have regarded kidney damage as a risk factor for acute and chronic myocardial injury (18–20). Cardiac troponin is a sensitive biomarker of myocardial injury and is often elevated above the conventional reference threshold in non-coronary artery diseases such as renal insufficiency (21), and the concentration of cardiac troponin in patients with kidney dysfunction is 3 times that of patients with normal kidney function (22). These results are consistent with this study, proving that hs-cTnT, as a marker of myocardial injury, has an indirect correlation with chronic kidney disease, but the direct correlation between hs-cTnT and chronic kidney disease warrants further investigation.

This study cannot determine the temporality of the association, and future cohort studies are needed to assess the changes in the relationship between hs-cTnT and lower limb disease over time. Monofilament testing is not the gold standard for PN, and PN diagnosed by monofilament testing represents the loss of protective sensation, which is a more severe form of PN. Nerve conduction studies or monofilament testing combined with ankle reflex and vibration sensation tests are more sensitive in diagnosing PN (23). Therefore, the prevalence of PN reported in our study may be underestimated. Currently, there is no exact incidence of cardiovascular diseases such as heart failure in NHANES, so this study adjusted for cardiovascular risk factors such as hypertension and hypercholesterolemia, but it is not possible to completely remove the interference of cardiovascular diseases to study the association between hs-cTnT and lower limb disease. The advantages of our study include a large sample size, measurement of hs-cTnT in diabetic patients across the United States, and strict measurement of traditional risk factors for lower limb disease by trained technicians using standardized protocols, including standardized monofilament testing for PN and ABI assessment for PAD. Also, we attempted to control for confounding variables through multivariate adjustment and subgroup analyses, including the use of medication.

In summary, hs-cTnT is closely associated with lower limb disease in adult diabetic patients. Our results suggest the utility of hs-cTnT as a biomarker for lower limb disease in adult diabetic patients and emphasize the potential direct correlation between hs-cTnT and chronic kidney disease.

## Data Availability

The original contributions presented in the study are included in the article/supplementary material. Further inquiries can be directed to the corresponding author/s.
